# The Latest

**Published:** 1902-03

**Authors:** 


The latest.—After our forms are closed, comes a press dispatch that Dr. I. J. Jones has been arrested in Tennessee. A detective has gone on from Austin to identify him and bring him back.

